# Porous Biomass Carbon Derived from *Clivia miniata* Leaves via NaOH Activation for Removal of Dye

**DOI:** 10.3390/ma15041285

**Published:** 2022-02-09

**Authors:** Wei Gao

**Affiliations:** College of Landscape Architecture, Changchun University, Changchun 130000, China; gaow@ccu.edu.cn

**Keywords:** biomass carbon, *Clivia miniata*, wastewater treatment, adsorption

## Abstract

*Clivia miniata* (CM), is an important ornamental plant and has been widely cultivated all over the world. However, there are no reports on *Clivia miniata*-based porous biomass carbon (CMBC). In this study, for the first time, CM leaves were used to generate porous biomass carbon via NaOH activation. The structures and surface characteristics were determined using scanning electron microscopy, N_2_ adsorption/desorption, TGA, FT-IR, X-ray diffraction, Raman and X-ray photoelectron spectra tests. CMBC has a large SSA (2716 m^2^/g) and a total pore volume of 1.95 cm^3^/g. To test the adsorption performance via adsorption experiments, the cationic and synthetic dye, malachite green (MG), was utilized as the adsorption model. The CMBC had a greatest adsorption capacity of 2622.9 mg/g at a pH value of 8 and had a fastest adsorption capacity of 1161.7 mg/g in the first 5 min. To explain MG adsorption into CMBC, the Freundlich isotherm and the pseudo-second-order kinetic model were used. The adsorption mechanism of MG was also investigated. After 10 cycles, the adsorption efficiency of CMBC to MG could still reach 85.3%. In summary, CMBC has excellent potential in dyeing wastewater pollution treatment.

## 1. Introduction

With the rapid development of industry, the demand for environmental pollution treatment is increasing [1,2,3]. Chemical flocculation, membrane filtration, ion exchange, oxidation processes, and adsorption processes are the most commonly used methods for pollution treatment today [4,5,6]. Adsorption is one of the most popular methods due to the advantages of easy operation, mildness, and high efficiency [5]. Generally, the adsorbent is considered a critical component in the adsorption process. Because of its porous structure and large surface area, biomass carbon produced by pyrolysis of biomass at high temperatures without or with limited oxygen content, has become one of the most commonly used adsorbents. It is also better than traditional carbon materials in terms of reproducibility and lower cost. So far, a large variety of waste biomass, such as coffee grounds, wood chips, husks, and straw, has been used as a raw material for the production of carbon materials [7,8,9,10]. There are two types of biomass carbon activation methods: physical activation and chemical activation. The physical activation method refers to the production of biomass carbons through the use of activators, such as water vapor, carbon dioxide, and a mixture of gases [11]. The chemical activation method refers to the use of NaOH, KOH, phosphoric acid, ZnCl_2,_ and other chemical reagents as activators to produce biomass carbons [12]. The physical activation method has advantages over the chemical activation method in terms of no secondary pollution to the environment and less corrosion to the equipment, but the required activation temperature is high, the activation time is long, and the prepared activated carbon pore structure area is low [12]. As a result, obtaining biomass-derived carbon materials with larger surface areas and improved adsorption properties is still a challenge.

Natural organisms have always attracted people because of their distinctive geometric structures. For decades, materials scientists have led the charge in developing intriguing materials and architectures inspired by nature. Our interest in this area has been piqued by the discovery of materials with unique natural structures and outstanding qualities [13,14,15,16]. *Clivia miniata* (CM), is an important ornamental plant and has been widely cultivated all over the world due to its unique shape and air purification properties [17]. The CM root is fleshy and fibrous, the leaves are dark green and leathery, with the leaf base forming false bulbs. It has sword-like leaves, up to 45 cm long, in an alternate arrangement, and produces whole terminal umbels, with 7 to 30 flowers per inflorescence. The small flowers are stalked and arranged in an umbrella at the top of the flower, funnel-shaped and erect. CM can flower all year round, but mainly in the spring and summer. Berries produced are globose, each fruit containing one or more seeds. The fruit ripening period is around October [18]. Studies on plant disease resistance and genomics of CM have recently attracted wide attention. There are no reports on *Clivia miniata*-based porous biomass carbon (CMBC), as far as we know. Thus, it is extremely important to investigate CMBC preparation technologies to increase their added value and to effectively utilize resources.

In this investigation, CM, with characteristic natural pores and a continuous long fiber structure, was used as the raw material. The CMBC preparation conditions were optimized. A variety of analyses were performed to test the features of CMBC manufactured under various conditions. Furthermore, as an adsorption model, a synthetic dye, malachite green (MG), was used, and the effects of pH, adsorption kinetics, adsorption isotherms, and adsorption thermodynamics were investigated. This study was conducted with a view to obtaining adsorption materials with excellent performance properties and to provide new ideas for the reuse of biomass.

## 2. Materials and Methods

### 2.1. Materials

CM was obtained from an experimental field of Changchun University in the autumn of 2021. Analytical pure grade hydrochloric acid, sodium hydroxide and the synthetic dye, malachite green (MG), without further treatment, were bought from Aladdin Chemical Co., LTD. (Shanghai, China).

### 2.2. Preparation of CMBC

The preparation conditions of CMBC were chosen according to the literature [19,20,21,22]. Dried CM was carbonized at 500 °C for 60 min under a nitrogen environment (0.1 L/min) with a heating rate of 10 °C/min after being crushed through a 100-mesh sieve. Carbonized CM (CCM) was mixed thoroughly with an activator (NaOH solid) at a ratio of 4:1 and heated at 700 °C for 30 min under a nitrogen environment (0.1 L/min). The sample was washed with HCl and deionized water to remove any excess alkali or carbonate. Different operating conditions, including different activation temperatures (600, 700, and 800 °C), impregnation ratios (3:1, 4:1, and 5:1), and activation times (15, 30, and 45 min), were investigated to test the effect of preparation conditions on sample performance. The carbonaceous materials were labeled according to the operating conditions, e.g., “CMBC-4-700-30” means that the CMBC was synthesized at 700 °C for 30 min with a NaOH/CCM ratio of 4:1.

### 2.3. Characterization

On a SHIMADZU SSX-550 microscope (SHIMADZU, Kyoto, Japan), scanning electron microscopy tests were carried out. At 77 K, nitrogen adsorption/desorption isotherms were measured using a Micromeritics ASAP 2020 adsorption analyzer (Micromeritics, Norcross, GA, USA). The specific surface area (SSA) was determined using the Brunauer–Emmett–Teller method [23]. Non-local density functional theory (NLDFT) was used to determine the pore size distribution [24]. The KBr tablet approach was used to obtain Fourier transform infrared (FT-IR) patterns with a resolution of 1 cm^−1^ between 400 and 6000 cm^−1^ using a Bruker IFS 66V/S spectrometer (Bruker, Hamburg, Germany). A Bruker D8 diffractometer (Bruker), with a Cu-K X-ray source, was used to measure the powder X-ray diffraction (XRD) pattern. An inVia Raman spectrometer (Renishaw 2000, Renishaw, Gloucestershire, UK), with a laser wavelength of 488 nm, was used to analyze the Raman properties of the carbon sample. X-ray photoelectron spectra were measured on a Thermo Escalab 250 electron energy spectrometer (Thermo Fisher, Waltham, MA, USA).

### 2.4. Adsorption Studies

In an equilibrium adsorption experiment, 10 mg of CMBC was inserted in a flask containing 100 mL of dye solutions. In a 303 K constant-temperature shaker, the mixture was shaken at 150 rpm. The suspension was obtained and centrifuged for 10 min at a speed of 12,000 rpm. The concentration was then determined using a UV-vis spectrophotometer (Agilent Cary300, USA) at the maximum wavelength of MG (λmax = 618 nm). The adsorption capacity (*Q_e_*, mg/g) was calculated by Equation (1):

(1) $$Q_{e}=\frac{\left(C_{0}-C_{e}\right)\times V}{m}$$

where *C_e_* (mg/L), *C*_0_ (mg/L), *V* (L) and m (g) represent the equilibrium concentrations of solutions, the initial concentration, the volume of the solutions, and the weight of the material, respectively. Each experimental run was repeated three times in parallel, and the average value was calculated.

The adsorption kinetics of MG onto CMBC were studied at different initial concentrations (50, 150, and 250 mg/L). The rate of adsorption kinetics were determined by fitting three of the adsorption kinetic models to the obtained adsorption data. Lagergren’s pseudo-first-order equation is the first model known to describe adsorption rate using adsorption capacity [25]. The pseudo-second-order model assumes that adsorbent and adsorbate share or exchange electrons during chemical adsorption [26].

Pseudo-first-order kinetic (Equation (2)) [25]:

(2) $$Q_{t}=Q_{e}\left(1-e^{-k_{1}t}\right)$$

Pseudo-second-order kinetic (Equation (3)) [26]:

(3) $$Q_{t}=\frac{k_{2}Q_{e}^{2}}{\left(1+k_{2}Q_{e}t\right)}t$$

Intra-particle diffusion (Equation (4)) [27]:

(4) $$Q_{t}=k_{3}t^{0.5}+C$$

where *Q_e_* (mg/g) is the adsorption capacity at equilibrium, *Q_t_* (mg/g) is the adsorption capacity at various time points, *C* (mg/g) is a constant that describes the thickness of the boundary layer, *k_1_* is the pseudo-first-order rate constant, *k_2_* is the pseudo-second-order rate constant, and *k_3_* is the intra-particle diffusion rate.

To study the adsorption isotherm of MG onto CMBC, 10 mg of CMBC was placed in flasks that contained solutions of different concentrations (50, 100, 150, 200, and 250 mg/L). The concentrations of the solutions were measured after 2 h. Then, the adsorption isotherm models were fitted to the obtained adsorption data using Langmuir and Freundlich isotherm models. The Langmuir isotherm model assumes that the adsorbate forms a monolayer on the adsorbent surface, whereas the Freundlich isotherm model was used for heterogeneous surface adsorption induced by a variety of surface functional groups.

Langmuir isotherm model (Equation (5)):

(5) $$Q_{e}=\frac{Q_{m}K_{L}C_{e}}{1+K_{L}C_{e}}$$

Freundlich isotherm model (Equation (6)):

(6) Qe=KFCe1nF

where *Q_m_* (mg/g) is the maximum adsorption capacity, *n_F_* is the Freundlich exponent, *K_L_* is the Langmuir constant, and *K_F_* is the Freundlich constant.

### 2.5. Effect of Temperature and pH

The effect of temperature on the adsorption capacity of CMBC was studied (*V*: 100 mL; *C*_0_: 400 mg/L) at different initial temperatures (293, 303, and 313 K). The concentrations of the solutions were measured after 2 h. To describe the effect of temperature on the adsorption process, thermodynamic parameters, such as standard free Gibbs energy (Δ*G*), standard enthalpy (Δ*H*), and standard entropy (Δ*S*) were calculated. Δ*G*, Δ*H*, and Δ*S* can be calculated by following Equations (7) and (8):

(7) ΔG=ΔH−TΔS

(8) $$\ln(\frac{Q_{e}}{C_{e}})=\frac{\Delta S}{R}-\frac{\Delta H}{RT}$$

where *Q_e_* and *C_e_* (mg/L) are the adsorption capacity of CMBC and the equilibrium dye concentration in solution, respectively. *T* is the temperature (K), and *R* represents the gas constant (8.3145 J/mol·K).

The effect of initial pH (2, 4, 6, 8, and 10) on the adsorption capacity of CMBC was studied (*V*: 100 mL; *C*_0_: 400 mg/L). The solutions were adjusted by using NaOH or HCl to different pH values. After 2 h, the concentration was measured.

### 2.6. Reusability Studies

The reusability of CMBC was explored through cycle adsorption tests. An amount of 0.1 g of CMBC was added to the MG solution (*V*: 100 mL; *C*_0_: 200 mg/L) in a cycle experiment [19,22]. After 2 h, the samples were separated by centrifuge at a speed of 12,000 rpm. The separated materials were washed and dried, then re-carbonized at 500 °C for 60 min under a nitrogen environment (0.1 L/min) with a heating rate of 10 °C/min and utilized as an adsorbent in subsequent cycles.

## 3. Results and Discussion

### 3.1. Characterizations

**Figure 1:**
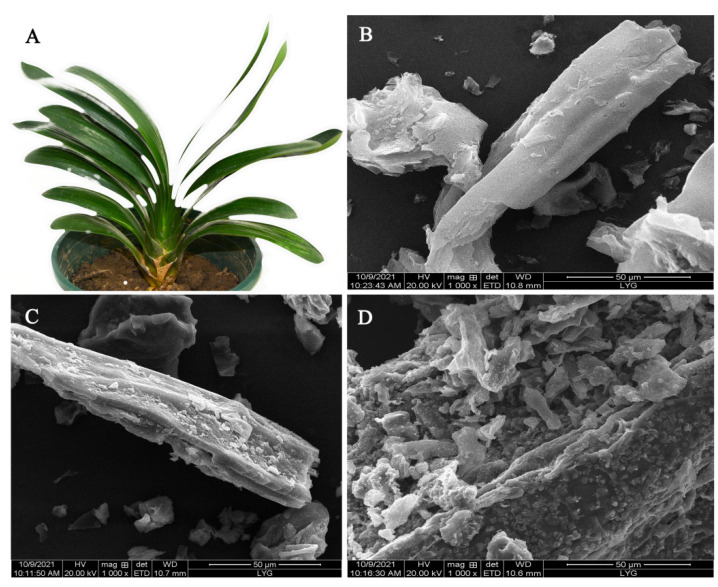
(**A**) A digital photo of CM and SEM images of (**B**) CM, (**C**) CCM, and (**D**) CMBC.

Figure 1A shows a digital photo of a CM plant with large, long fibrous leaves. The morphologies of CM, CCM, and CMBC were studied and are depicted in Figure 1B–D, respectively. The surface of CM was smooth and regular, with a filiform structure, as seen in Figure 1B. The structure of CCM became rough after carbonization (Figure 1C), showing that the sample was successfully dehydrated during this procedure and that carbonization did not disrupt the CM filamentous structure [19]. The surface of the CMBC, however, formed a large number of broken structures following activation, as seen in Figure 1D, indicating that the high temperature and activator did indeed activate CCM [20,21,22].

TGA was used to examine the thermal stability and component loss rate of materials (Figure S1). The TGA curve showed a slight weight loss in the first thermal breakdown stage, which ranged from the starting temperature to 200 °C. At this stage, water and gas adsorbed were liberated from the CM’s interior pores and surface [19]. The greatest weight loss occurred in the second thermal breakdown stage (200–500 °C) and may have been caused by the disintegration of oxygen-containing components [22]. The TGA curve gradually dropped at 500 °C, and CM weight loss remained stable. As a result, a carbonization temperature of 500 °C was chosen.

**Figure 2:**
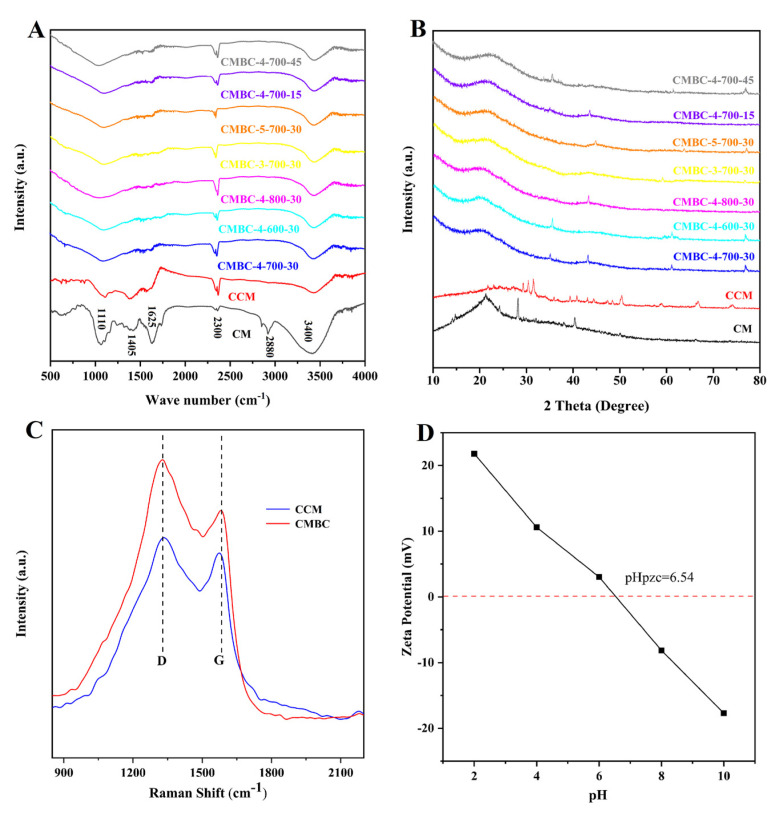
(**A**) FT-IR, (**B**) XRD tests of CM, CCM and CMBC prepared at different conditions, (**C**) Raman tests of CCM and CMBC-4-700-30 and (**D**) zeta potential test of CMBC-4-700-30.

The surface chemical characteristics, crystalline degree, and defects in carbon materials were investigated using FT-IR (Figure 2A), XRD (Figure 2B), and Raman spectroscopy (Figure 2C), respectively. As shown in the FT-IR results (Figure 2A), the band peaks around 3400 cm^−1^ correspond to the -OH groups stretching [21,22]. The C-H stretch [28] was associated with the band peaks around 2880 cm^−1^. The band peaks around 2300 cm^−1^ were attributed to the cyanogroup or carbon dioxide [28]. The carbonyl group underwent axial deformation in the region of 1610–1650 cm^−1^ [29]. The band peak at 1405 cm^−1^ was attributed to C-C [30]. The peaks around 1105–1140 cm^−1^ were attributed to the C-O functional group [31]. The C-C bands vanished after activation, which could explain why the majority of the C-C bands originated from proteins and polysaccharides in CM, which would react with NaOH during the high-temperature pyrolysis [20,22]. The functional groups of CMBC prepared at different conditions did not differ greatly. CCM and CMBC, like many biomass carbons [32,33], contained two major constituents following carbonization and activation: C and O (Figure S2).

According to the XRD pattern (Figure 2B), the samples had a peak at 21.5°, which corresponded to the cellulose peak [34]. Furthermore, the results show that there were some small and sharp diffraction peaks in CCM, which can be interpreted as inorganic salts and the salting-out effect caused by water loss or CM cracking. When comparing CCM and CMBCs to CM, the peak intensity of 21.5° continued to fade, possibly because the carbonization and activation processes produced more amorphous carbon [35]. In addition, it was found that the crystal structure of CMBCs, prepared at different temperatures and times, was not significantly different. CCM contained two primary distinctive peaks at 1350 cm^−1^ (i.e., D peak, amorphous carbon) and 1550 cm^−1^ (i.e., G peak, crystalline graphite), as revealed in the Raman spectra (Figure 2C). The I_D_/I_G_ (intensity ratio between amorphous carbon and crystalline graphite) increased from 1.32 to 1.86, indicating that the crystalline graphite content in CMBC-4-700-30 decreased as well [36]. The crystalline graphite content decreased after activation, as it does for many other types of biomass carbon [19,20,21,22]. Furthermore, the finding was consistent with the XRD test results.

The electrical properties of adsorbent surfaces are often measured by the zeta potential [37]. The variable surface charge and the permanent charge determine the point of zero charges, which is usually referred to as the proton interaction charge density, the permanent charge density, the charge density of the internal coordination complex, and the corresponding pH (pH_PZC_), when the sum of the charge density of the external coordination complex is zero [37]. It can be seen from the results of the zeta potential test of CMBC-4-700-30 (Figure 2D) that with pH value increase from 2 to 10, the value of the zeta potential changed from positive to negative, which meant the CMBC surface charge became negative. The pH_PZC_ was 6.54, which indicates that CMBC is better suited for the treatment of cationic contaminants in higher pH environments.

**Figure 3:**
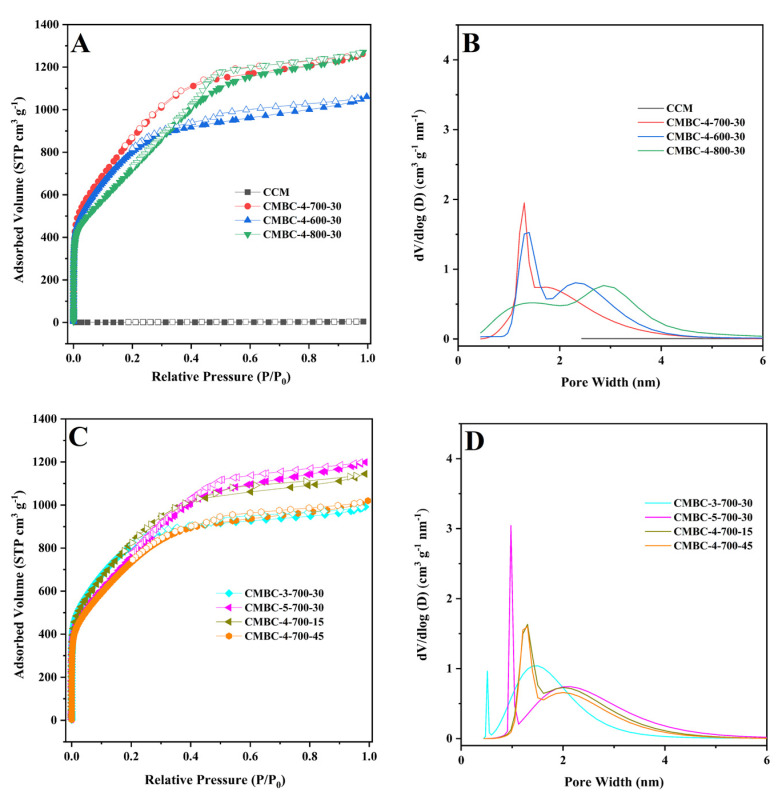
(**A**) and (**C**) N_2_ adsorption-desorption isotherms of CCM, CMBC-4-700-30, CMBC-4-600-30, and CMBC-4 800-30, CMBC-3-700-30, CMBC-5-700-30, CMBC-4-700-15, and 267 CMBC-4-700-45 and (**B**) and (**D**) their pore size distribution.

The N_2_ adsorption-desorption isotherm was used to investigate the SSA and pore properties of CCM and CMBC produced under various circumstances (Table 1 and Figure 3). As illustrated in Figure 3A,C, CMBC under various preparation conditions exhibited typical type-I or type-IV isotherms [38,39], indicating that CMBC possessed an excess of mesoporous characteristics. CMBC under various preparation circumstances not only contained a considerable number of mesopores, but also a large number of micropores, as seen in Figure 3B and 3D. CCM had an SSA of 2.6 m^2^/g, but CMBCs had significant SSAs of 2716, 2759, 2354, 2772, 2468, 2689, and 2429 m^2^/g, implying that the large SSA was due to activation rather than carbonization. The possible mechanism of the reaction of NaOH with CCM [21,22,39,40]:

$$2\mathrm{NaOH}\to {\mathrm{Na}}_{2}O+H_{2}O$$

$$C+H_{2}O\to H_{2}+CO\;$$

$$\mathrm{CO}+H_{2}O\to H_{2}+CO_{2}\;$$

$${\mathrm{Na}}_{2}O+{\mathrm{CO}}_{2}\to Na_{2}CO_{3}\;$$

When using NaOH as an activator, it is decomposed into cation (Na^+^) and hydroxyl anion (OH^-^) during high-temperature pyrolysis. During activation, the ions react with water to embed or migrate into the carbon structure, forming carbonates. These reactions result in the formation of pore structures in the acid and washed carbon [39,40]. The SSA of CMBC-4-600-30 and CMBC-4-800-30 was lower than CMBC-4-700-30, as indicated in Table 1, possibly due to insufficient activation at low temperature and excessive activation at high temperature [40]. When the SSA of CMBC-4-700-15, CMBC-4-700-45, and CMBC-4-700-30 are compared, it is possible to conclude that an incomplete reaction with short reaction time and excessive material activation with long reaction time occurred [40]. The SSAs of CMBC-3-700-30 and CMBC-5-700-30 were 2772 and 2468 m^2^/g, respectively, demonstrating that the activator-to-CCM ratio may influence the SSA. The NLDFT method was used to investigate the pore distribution. Table 1 shows that the pore diameters of CMBCs were less than 2 nm under various conditions, with the majority concentrated at 0.7 ± 0.05 nm, indicating that microporous structures make up the majority of CMBCs. The micropore surface areas were 1755, 1001, 348, 1958, 722, 1282, and 1224 m^2^/g and the micropore volume also occupied the majority of the total pore volume. To summarize, the CMBCs were microporous materials with partial mesoporous structures. The CMBC microporous structures allow for rapid adsorption in a water environment [22,41,42]. Furthermore, as indicated in Figure S3, CMBC-4-700-30 had a larger adsorption capacity than the others. Although the SSA of CMBC-3-700-30 was slightly larger than that of CMBC-4-700-30, its adsorption capacity was not as high as that of CMBC-4-700-30, which can be interpreted as the total pore volume of CMBC-4-700-30 (1.95 cm^3^/g) being higher than that of CMBC-3-700-30 (1.53 cm^3^/g). Moreover, the micropore volume in CMBC-3-700-30 (0.91 cm^3^/g) was higher than that in CMBC-4-700-30 (0.69 cm^3^/g). Thus, CMBC-4-700-30, prepared at an activation temperature of 700 °C, with an activation time of 30 min, and a ratio of alkali to carbon of 4:1, was chosen as the model CMBC for the following adsorption experiments.

### 3.2. Adsorption Experiments

#### 3.2.1. Adsorption Kinetics

**Figure 4:**
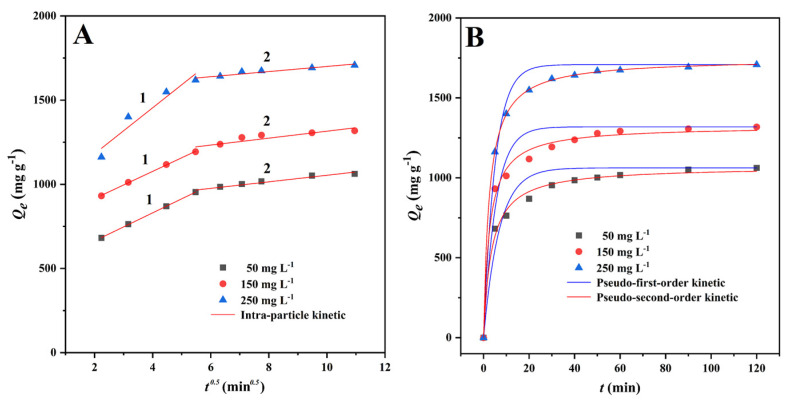
Non-linear fitting of MG adsorption kinetics onto CMBC-4-700-30 at different initial concentration at 303 K. (**A**) Intra-particle diffusion model and (**B**) pseudo-first-order and pseudo-second-order kinetics.

The adsorption rate is a critical metric for assessing the adsorption process [43]. As can be seen from the results, the adsorption capacity grew rapidly in the first 5 min, and the adsorption curve followed suit, reached adsorption equilibrium within 1 h (Figure 4B). This may have been due to the adsorption sites on the CMBC-4-700-30 surface becoming saturated and reaching adsorption-desorption equilibrium.

As can be seen from Figure 4A, when the intra-particle diffusion model was used to describe the MG adsorption onto CMBC-4-700-30, single linearity was not observed in this study. As in some previous studies [40,44], the MG adsorption onto CMBC-4-700-30 could be divided into three processes with different adsorption rate constants. At the first time interval (0–5 min), the fast adsorption is due to the abundant adsorption sites on the surface of CMBC-4-700-30. At the second time interval (5-30 min), the *k_3-1_* values were 83.39, 80.80, and 136.65 mg g^−1^ min^−1^. The adsorption rate *k_3-2_* values were significantly less than those for the second time interval. Compared with the second time interval, the adsorption amount in the time interval 30–120 min increased slowly, indicating that only a small amount of the MG was removed from solution by diffusion into internal channels and vacancies. The *R^2^* of the kinetic models was 0.9367–0.9810 for the pseudo-first-order kinetic model and 0.9927–0.9999 for the pseudo-second-order kinetic model. It can be easily seen that the pseudo-second-order model has a very good fitting effect, as evidenced by the high correlation value (*R^2^* > 0.99) and fitting plots (Figure 4B). The values of *Q_e_* calculated from pseudo-second-order models (1070.8, 1323.9, and 1745.4 mg/g) were slightly higher than the experimentally observed values (1051.5, 1306.5, and 1692.1 mg/g, Table 2). These findings suggest that the pseudo-second order model was better suited to describe the MG adsorption onto CMBC-4-700-30. As a result, the adsorption kinetics of CMBC-4-700-30 to MG were modeled using a pseudo-second-order kinetics model, revealing that chemical adsorption was involved in the process [45]; these results were similar to those of previous studies [46,47,48].

#### 3.2.2. Adsorption Isotherms

**Figure 5:**
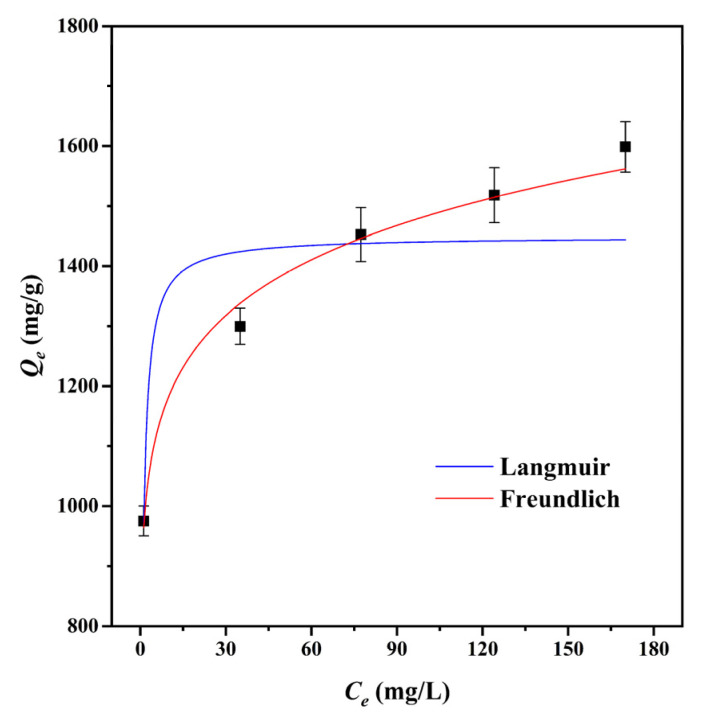
Adsorption isotherms of CMBC-4-700-30 to MG at 303 K.

The adsorption isotherm is a significant factor in the optimization of the adsorption mechanism since it defines the interaction between adsorbates and adsorbents [49]. The Langmuir and Freundlich isotherm models were used to fit the adsorption data of CMBC-4-700-30 to MG at 303 K, as shown in Table 3 and Figure 5. We can see from the data for the Langmuir adsorption isotherm that the value of the maximum adsorption capacity (*Q_m_*) of CMBC-4-700-30 was 1483.1 mg/g, which indicates that CMBC-4-700-30 had a high adsorption capacity for MG. The *K_F_* of CMBC-4-700-30 was 936.2 and the intensity factor n was 9.84. The surface uniformity of adsorbent is denoted by 1/*n* value and the surface becomes more nonuniform as 1/*n* approaches zero [22]. When *n* is 9.8425, 1/*n* is 0.1016, indicating favorable adsorption on the surfaces of multilayer and heterogeneous adsorbents. The correlation coefficients (*R^2^*) of the Langmuir isotherm model and the Freundlich isotherm model were 0.8296 and 0.9888, respectively. The results indicated that the Freundlich isotherm model may better fit the adsorption of CMBC-4-700-30 to MG compared to the Langmuir isotherm model. Furthermore, this also implies that MG adsorbed on CMBC-4-700-30 may have a heterogeneous surface and multilayer adsorption [50].

### 3.3. Effect of Temperature and pH

**Figure 6:**
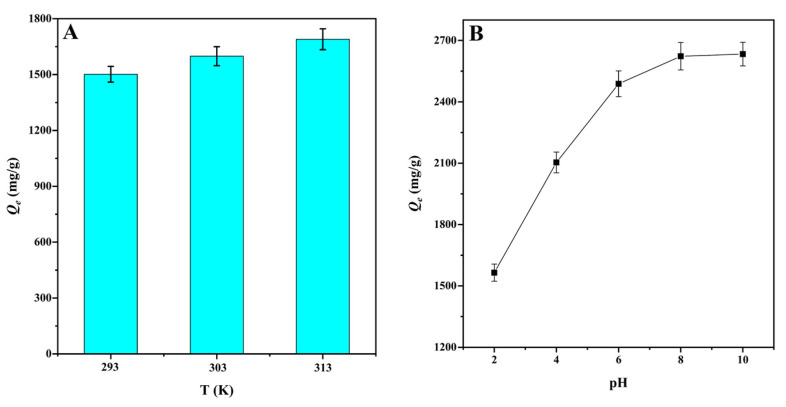
(**A**) The effect of temperature on the adsorption capacities for MG at natural pH value (*V*: 100 mL; *C*_0_: 400 mg/L; natural pH: 3.8 ± 0.2) and (**B**) the effect of pH on the adsorption capacities of MG at 303 K (*V*: 100 mL; *C*_0_: 400 mg/L).

The solution temperature is considered to be an important factor affecting adsorption equilibrium [51]. With temperature increase from 293 to 303 K, the adsorption capacity for MG adsorption onto CMBC-4-700-30 increased from 1501.8 to 1598.7 mg/g. By raising the temperature to 313 K, the adsorption was enhanced to 1689.8 mg/g. This can be explained, on the one hand, by the fact that increasing temperature increases molecular thermal motion in the solution. On the other hand, the higher temperature promotes the adsorption driving force, and thus increases the adsorption capacity [22]. The results indicate that temperature increase might promote the adsorption process (Figure 6A). Thermodynamic parameters were used to evaluate the adsorption process, including the standard Gibbs free energy (Δ*G*), the standard enthalpy (Δ*H*), and standard entropy (Δ*S*) (Table 4). The negative values of Δ*G* at various temperatures indicated that the adsorption of MG for CMBC-4-700-30 was spontaneous and beneficial. With increase in temperature, the value of Δ*G* also increases, and the spontaneity of the reaction system increases, which can be interpreted that an increase in temperature will lead to an increase in the mobility of the dye from the solution to the adsorbent surface [52]. When *∆H* is less than 4.2 kJ mol^−1^, it indicates that adsorption tends to physical adsorption, and vice versa [22]. The Δ*H* (−5.62) was negative and greater than −4.2, suggesting adsorption onto CMBC-4-700-30 has endothermic properties and was chemical adsorption. A positive value of Δ*S* indicates that the process has a high disorder in the solid solution interaction, while a negative value indicates that the process has a low disorder. The calculated standard entropy (Δ*S*) was positive, indicating that, as temperature increased, so did the disorder and randomness of the solid/solution interface.

The pH value of the solution is an important factor affecting the adsorption capacity by changing the surface charge of the adsorbents [53]. Figure 6B shows the effect of pH on the adsorption capacity of MG onto CMBC-4-700-30. When the pH was less than pH_pzc_ = 6.54, the surface of the CMBC-4-700-30 was positively charged, and large hydrogen ions competed for adsorption sites with dye cations, potentially repelling the cationic dye and reducing adsorption capacity. When the pH of the solution was increased to pH_pzc_ = 6.54, the adsorption capability of CMBC-4-700-30 to MG increased dramatically. This is explained by the fact that as the pH of the solution decreased, the adsorbent’s negative charge increased, promoting electrostatic contact between the cationic dye and the adsorbent surface. When the pH was increased from pH_pzc_ = 6.54 to 8, however, the increasing trend of CMBC-4-700-30 adsorption capacity was slightly and slowly increased. Finally, with a pH value greater than 8, adsorption capacity was not increased, indicating that pH was not the only factor influencing adsorption performance [20]. Furthermore, the results revealed that CMBC-4-700-30 had a greater maximum adsorption capacity than most other adsorbents previously reported (Table S1), including *Borassus aethiopum* flower activated carbon, rice straw-derived char, magnetic graphene oxide, activated biochar derived from *Opuntia ficus-indica*, and so on. As a result, CMBC-4-700-30 might be considered a promising material for the treatment of dyeing wastewater pollution.

### 3.4. Reusability of the CMBC-4-700-30 and Probable Mechanism Analysis

**Figure 7:**
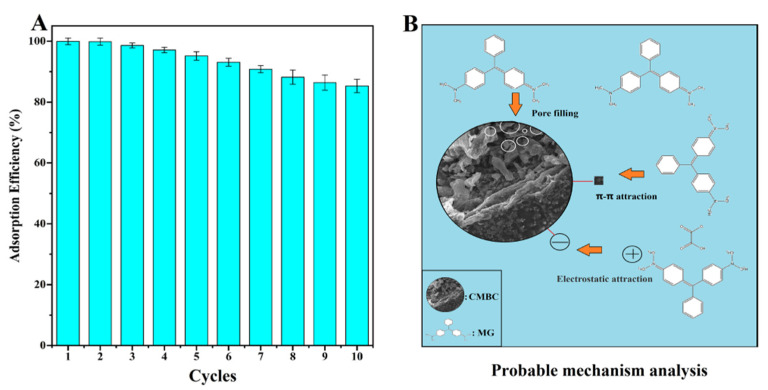
(**A**) The reusability and (**B**) probable mechanism analysis of CMBC-4-700-30 for removal of MG.

The reusability of CMBC-4-700-30 for MG removal was demonstrated in Figure 7A. The reusability of the adsorbent, which relates to the cost benefit of adsorbent, is an important indicator throughout the practical application phase [54]. Through further treatment of contaminants, the regeneration process can lessen the danger of secondary pollution. Repeated carbonization was used to eliminate adsorbent MG in preparation for the following cycle. The CMBC-4-700-30 still retained an 85.3% MG removal efficiency until 10 cycles were completed, which could be attributed to the deposition of byproducts on the surfaces of CMBC. The results indicate that CMBC-4-700-30 has high reusability for eliminating MG.

The pore filling, π-π interaction, and electrostatic attraction were all shown to be possible adsorption mechanisms of CMBC-4-700-30 for MG (Figure 7B). More pore filling sites provided by CMBC-4-700-30’s large SSA (2716 m^2^/g) and total volume (1.95 cm^3^/g) allow more dye molecules to mix with absorbent, potentially improving CMBC-4-700-30 MG adsorption capacity. Adsorption may be aided by the π-π-interaction between the bonds in the CMBC-4-700-30’s graphite structure and the aromatic ring of MG. Electrostatic interaction between the negatively charged CMBC-4-700-30, and the positively charged cationic dye MG, may occur as the pH is gradually increased, potentially improving adsorption performance. As a result, this high adsorption capacity and reusable adsorbent may have a bright future in the treatment of dye wastewater.

## 4. Conclusions

In this study, CM was used as a raw material for the first time in the manufacture of porous biomass carbon materials. The results of the characterization showed that the CMBC-4-700-30 has an enormous SSA of 2716 m^2^/g and a high pore volume of 1.95 cm^3^/g. The possible mechanism of the reaction of NaOH with carbon was explored. Furthermore, the experimental results demonstrated that the CMBC-4-700-30 could cope better for MG pollution treatment. The greatest adsorption capacity was reached at 2622.9 mg/g at a pH of 8, which was higher than for most other reported adsorbents, including many biomass activated carbon materials. After adsorption studies, the Freundlich isotherm and the pseudo-second-order adsorption kinetic model fitted the adsorption process very well, indicating that the adsorption of MG onto CMBC-4-700-30 occurred through chemical and multilayer adsorption. After 10 cycles, the adsorption efficiency of MG could still be as high as 85.3%. Moreover, the pore filling, π-π interaction, and electrostatic attraction might enhance the good adsorption performance of CMBC-4-700-30. In conclusion, CMBC-4-700-30 has good recyclability and high adsorption performance. In the future, we will explore further the application of CMBC-4-700-30 in other fields.

## Figures and Tables

**Table 1:** The data of N_2_ adsorption-desorption for CCM, CMBC prepared under different conditions.

Samples	Conditions			S_BET_ (m^2^/g)	S_Micro_ (m^2^/g)	V_Micro_ (cm^3^/g)	V_total_ (cm^3^/g)
	NaOH:Carbon	T (°C)	t (min)				
CCM	-	500	60	3	-	-	0.0062
CMBC-4-600-30	4:1	600	30	2759	1755	0.82	1.63
CMBC-4-700-30	4:1	700	30	2716	1001	0.69	1.95
CMBC-4-800-30	4:1	800	30	2354	348	0.39	1.96
CMBC-3-700-30	3:1	700	30	2772	1958	0.91	1.53
CMBC-5-700-30	5:1	700	30	2468	722	0.53	1.85
CMBC-4-700-15	4:1	700	15	2689	1282	0.73	1.77
CMBC-4-700-45	4:1	700	45	2429	1224	0.66	1.57

S_BET_ and S_Micro_ (m^2^/g) are the BET and micropore surface area; V_Micro_ and V_total_ (cm^3^/g) are micropore and the total pore volume.

**Table 2:** Fitting parameters of the adsorption kinetic models.

*C*_0_ (mg/L)	*Q_e_* (mg/g)	Pseudo-First-Order Kinetic			Pseudo-Second-Order Kinetic			Intra-Particle Diffusion			
		*k*_1_ (min^−1^)	*Q_e.cat_* (mg/g)	*R* ^2^	*k*_2_ (g mg^−1^ min^−1^)	*Q_e.cat_* (mg/g)	*R* ^2^	*k*_3-1_ (mg g^−1^ min^−1^)	*R* ^2^	*k*_3-2_ (mg g^−1^ min^−1^)	*R* ^2^
50	1051.5	0.1465	1061.8	0.9367	0.0003	1070.8	0.9932	83.39	0.9997	19.45	0.9509
150	1306.5	0.1874	1318.2	0.9428	0.0003	1323.9	0.9927	80.80	0.9992	20.62	0.8024
250	1692.1	0.1991	1707.9	0.9810	0.0002	1745.4	0.9999	136.65	0.9301	15.22	0.9160

**Table 3:** Fitting parameters of the Langmuir, Freundlich and Temkin isotherm models at 303 K.

Isotherm Models	Constants	
Langmuir	*Q_m_* (mg/g)	1483.1
	*K*_L_ (L/mg)	1.50
	*R^2^*	0.8296
Freundlich	*K_F_* (mg g^−1^(L mg^−1^)^1/n^)	936.2
	*n*	9.8425
	*R^2^*	0.9888

**Table 4:** Thermodynamic parameters for the adsorption of MG onto CMBC-4-700-30.

Temperature (K)	*Q_e_* (mg/g)	Δ*G* (kJ mol^−1^)	Δ*H* (kJ mol^−1^)	Δ*S* (J mol^−1^ K^−1^)
293	1501.8	−3.73	−5.62	13.36
303	1598.7	−4.05		
313	1689.8	−4.36		

## Data Availability

The data presented in this study are contained within the article.

## Supplementary Materials

The following are available online at https://www.mdpi.com/article/10.3390/ma15041285/s1: Figure S1: TGA and DTG curves of CM with the heating rate of 10 °C/min under the atmosphere of N_2_; Figure S2: XPS test of CCM and CMBC; Figure S3: The adsorption capacities of CMBC prepared at different conditions to MG (*T*: 303 K; Adsorbent: 10 mg; *C*_0_: 400 mg L^−1^; *V*: 100 mL); Table S1: Comparison of the adsorption capacities of samples to MG with other adsorbents.
