# Supplementary material for: Porous Biomass Carbon Derived from Clivia miniata Leaves via NaOH Activation for Removal of Dye
Source: Materials (Basel). 2022 Feb 9;15(4):1285. doi: 10.3390/ma15041285 (PMC8880077; doi:10.3390/ma15041285)
Supplement: Supplementary file 1 [file materials-15-01285-s001.zip › materials-1546138-supplementary.pdf]

Porous Biomass Carbon Derived From *Clivia miniata* Leaves via NaOH Activation for Removal of Dye

Wei Gao[1,*]

College of Landscape Architecture, Changchun University, Changchun 130000, China;

* Correspondence: gaow@ccu.edu.cn

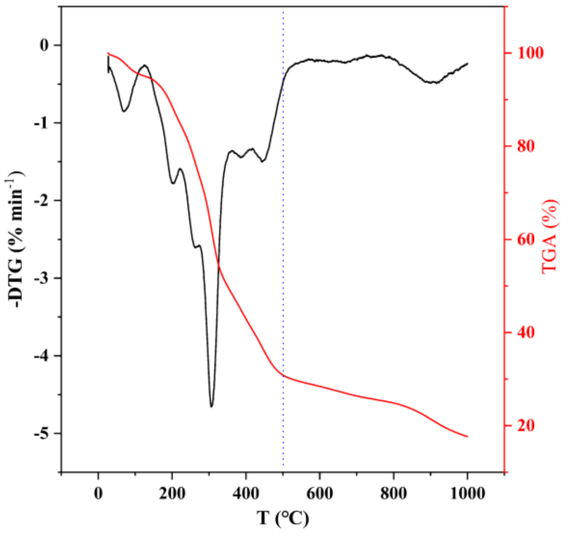

Figure S1. TGA and DTG curves of CM with the heating rate of 10 °C/min under the atmosphere of N_2.

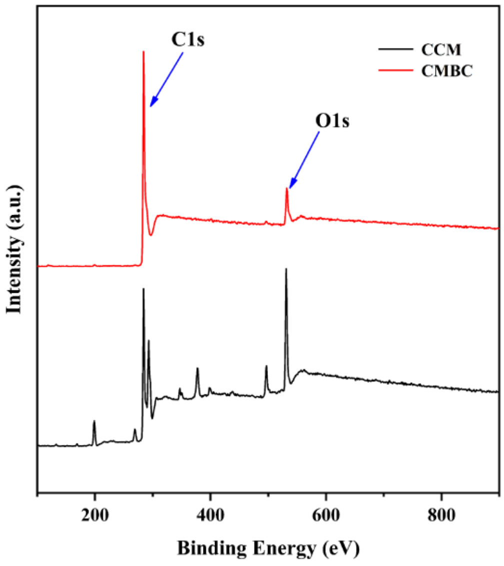

Figure S2. XPS test of CCM and CMBC.

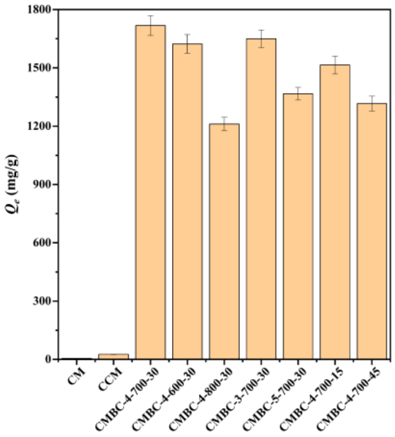

Figure S3. The adsorption capacities of CMBC prepared at different conditions to MG (T: 303 K; Adsorbent: 10 mg; C_0: 400 mg/L; V: 100 ml).

Table S1 Comparison of the adsorption capacities of samples to MG with other adsorbents.

Adsorbent	pH	Time	Temperature (K)	Q_e (mg/g)	References
Polylactide/spent brewery grains films	4.5	45 min	296	1.5	[1]
Brewers' spent grain	neutral	120 min	296	2.6	[2]
Neem sawdust	7.2	20 min	298	4.4	[3]
Chemically modified rice husk	7	120 min	298	12.2	[4]
Borassus aethiopum flower activated carbon	6.87	24 h	300	20.5	[5]
Clayey soil of Indian origin	6.0	100 min	303	78.6	[6]
Modified sphagnum peat moss	6.5	90	293	122.0	[7]
Rice straw-derived char	5	2 h	303	148.7	[8]

Sodium carboxymethyl cellulose aerogels	7	12 h	298	245.3	[9]
Activated carbon pellet	3.2	24 h	room temperature	395.0	[10]
nZVI/BC	6	10 min	313	515.8	[11]
AFA	9	12 h	323	637.6	[12]
Magnetic graphene oxide	7	30 min	298	714.3	[13]
TEMPO-oxidized cellulose beads	6.86	300 min	298	740.0	[14]
Activated graphene	neutra	40 min	298	791.3	[15]
Fibrous cellulose sulfate	neutra	30 min	298	960.0	[16]
Activated biochar derived from Opuntia ficus-indica	6	120 min	303	1341.0	[17]
Porous Carbon Material Based on Quinoa Husk	7	120 min	298	1365.1	[18]
CM	3.8	120 min	303	5.4	This work
CCM	3.8	120 min	303	26.2	This work
CMBC	8	120 min	303	2622.9	This work

References

[1] Chanzu, H.; Onyari, J.; Shiundu, P. Biosorption of malachite green from aqueous solutions onto polylactide/spent brewery grains films: kinetic and equilibrium studies. *J. Polym. Environ.* 2012, 20, 665-672.

[2] Chanzua, H.; Onyarib, J.; Shiundu, P. Brewers' spent grain in adsorption of aqueous Congo Red and malachite Green dyes: Batch and continuous flow systems. *J. Hazard. Mater.* 2019, 380, 120897.

[3] Khattri, S.; Singh, M. Removal of malachite green from dye wastewater using neem sawdust by adsorption. *J. Hazard. Mater.* 2009, 167, 1089-1094.

[4] Chowdhury, S.; Mishra, R.; Saha, P.; Kushwaha, P. Adsorption thermodynamics, kinetics and isosteric heat of adsorption of malachite green onto chemically modified rice husk. *Desalination.* 2011, 265, 159-168.

[5] Nethaji, S.; Sivasamy, A.; Thennarasu, G.; Saravanan, S. Adsorption of Malachite Green dye onto activated carbon derived from Borassus aethiopum flower biomass. *J. Hazard. Mater.* 2010, 181, 271-280.

[6] Saha, P.; Chowdhury, S.; Gupta, S.; Kumar, I. Insight into adsorption equilibrium, kinetics and thermodynamics of Malachite Green onto clayey soil of Indian origin. *Chem. Eng. J.* 2010, 165, 874-882.

[7] Hemmati, F.; Norouzbeigi, R.; Sarbisheh, F.; Shayesteh, H. Malachite green removal using modified sphagnum peat moss as a low-cost biosorbent: Kinetic, equilibrium and thermodynamic studies. *J. Taiwan Inst. Chem. E.* 2015, 58, 482-489.

[8] Hameed, B.; El-Khaiary, M. Kinetics and equilibrium studies of malachite green adsorption on rice straw-derived char. *J. Hazard. Mater.* 2008, 153, 701-708.

[9] Yu, M.; Li, J.; Wang, L. KOH-activated carbon aerogels derived from sodium carboxymethyl cellulose for high-performance supercapacitors and dye adsorption. *Chem. Eng. J.* 2017, 310, 300-306.

[10] Hui, S.; Zaini, T. Development of activated carbon pellets using a facile low-cost binder for effective malachite green dye removal. *J. Clean. Prod.* 2020, 253, 119970.

[11] Eltaweil, A.; Mohamed, H.; El-Monaem, M.; El-Subruiti, G. Mesoporous magnetic biochar composite for enhanced adsorption ofmalachite green dye: Characterization, adsorption kinetics,thermodynamics and isotherms. *Adv. Powder. Technol.* 2020, 31, 1253-1263.

[12] An, Y.; Zheng, H.; Zheng, X.; Sun, Q.; Zhou, Y. Use of a floating adsorbent to remove dyes from water: A novel efficient surface separation method. *J. Hazard. Mater.* 2019, 375, 138-148.

[13] Yakout, A.; Shaker, M. Dodecyl sulphate functionalized magnetic graphene oxide nanosorbent for the investigation of fast and efficient removal of aqueous malachite green. *J. Taiwan Inst. Chem. E.* 2016, 63, 81-88.

[14] Lin, F.; You, Y.; Yang, X.; Jiang, X.; Lu, Q.; Wang, T.; Huang, B.; Lu, B. Microwave-assisted facile synthesis of TEMPO-oxidized cellulose beads with high adsorption capacity for organic dyes. *Cellulose.* 2017, 24, 5025-5040.

[15] Kim, M.; Sharma, N.; Chung, J.; Yun, K. Activated graphene with fractal structure for the adsorption of malachite green with high removal rate. *Micropor. Mesopor. Mat.* 2021, 322, 111166.

[16] Baghdadi, M.; Soltani, B.; Nourani, M. Malachite green removal from aqueous solutions using fibrous cellulose sulfate prepared from medical cotton waste: Comprehensive batch and column studies. *J. Ind. Eng. Chem.* 2017, 55, 128-139.

[17] Choudhary, M.; Kumar, R.; Neogi, S. Activated biochar derived from Opuntia ficus-indica for the efficient adsorption of malachite green dye, Cu^{+2} and Ni^{+2} from water. *J. Hazard. Mater.* 2020, 392, 122441.

[18] Chen, S.; Tang, S.; Sun, Y.; Wang, G.; Chen, H.; Yu, X.; Su, Y.; Chen, G. Preparation of a Highly Porous Carbon Material Based on Quinoa Husk and Its Application for Removal of Dyes by Adsorption. *Materials.* 2018, 11, 1407.
